# Infectivity to *Phlebotomus perniciosus *of dogs naturally parasitized with *Leishmania infantum *after different treatments

**DOI:** 10.1186/1756-3305-4-52

**Published:** 2011-04-13

**Authors:** Guadalupe Miró, Rosa Gálvez, Cristeta Fraile, Miguel A Descalzo, Ricardo Molina

**Affiliations:** 1 Departamento de Sanidad Animal, Facultad de Veterinaria, Universidad Complutense, Madrid, Spain; 2 Servicio de Parasitología, Centro Nacional de Microbiología, Instituto de Salud Carlos III, Majadahonda, Madrid, Spain; 3 Unidad de Investigación, Fundación Española de Reumatología, Madrid, Spain

## Abstract

**Background:**

In Europe most dogs with clinical leishmaniosis are treated with leishmanicides, typically antimonials combined with allopurinol and good clinical recovery is observed in a high number of these dogs. Through xenodiagnosis, the capacity of a treated animal to infect the vector of the disease under treatment is assessed as a measure of the chemotherapeutic efficacy of the drug used. The objective of the present study was to evaluate through direct xenodiagnosis the infectivity to *Phlebotomus perniciosus *of dogs naturally parasitized with *Leishmania infantum *after treatment, and to follow the clinical and parasite course of disease. Thirty two dogs with clinical leishmaniosis were assigned to one of three treatment groups: meglumine antimoniate plus allopurinol (Group A), meglumine antimoniate (Group B) or allopurinol (Group C). During the study, the dogs were examined before treatment (Day 0) and bimonthly thereafter until Day 180 (six months post-treatment onset).

**Results:**

The three groups were scored over time according to the effects of treatment on clinical signs and clinical-pathological variables. Significant differences in clinical scores were observed between Group A and the other two groups, indicating the combined treatment was the most effective. After treatment, bone marrow cultures were positive for the parasite in 30.8% of dogs in some of the check ups (3 or 25% in Group A, 1 or 11.1% in Group B, and 4 or 80% in Group C). Our xenodiagnosis experiments revealed that 15.4% of treated dogs were still able to infect sand flies at some point after treatment (2 dogs or 16.6% in Group A, 2 or 22.2% in Group B and none in Group C). Only 7.7% of the entire study population could infect sand flies as from the second month post-treatment onset.

**Conclusion:**

The three treatment regimens tested significantly reduced the infectivity of dogs towards sand flies, thus diminishing the epidemiological risks of treated dogs both for human beings and other healthy dogs. Despite its low cure rate, the use of allopurinol after a course of leishmanicide treatment is proposed to keep dogs non-infectious during the disease transmission season (4-6 months in southern Europe).

## Background

Leishmaniosis caused by the protozoan *Leishmania infantum *is a severe parasitic disease that is spread to humans and animals by blood-sucking phlebotomine sand flies. Leishmaniosis is endemic in the Mediterranean basin. In Spain, the proven vectors for *L. infantum *are *Phlebotomus perniciosus *and *Phlebotomus ariasi *[1-3], the former being the main vector. Dogs are the main domestic reservoir of the parasite and play a role in human infection [4]. Notwithstanding, the epidemiological role of the cat is today a subject of intense research [5-10] and it has been confirmed through direct xenodiagnosis that cats presenting clinical signs of the disease are able to infect sand flies [11,12].

Canine leishmaniosis (CanL) is today one of the most important imported canine diseases in Central Europe [13]. Estimates of CanL seroprevalence reported for Spain range from 3.7% for the Orense province in the northwest corner of the country [14] to 34.6% for the province of Málaga on the south coast [15]. In the Madrid region, a significant increase in the seroprevalence of CanL (from 5.25% to 8.1%) and the densities of both its vectors (*P. perniciosus *and *P. ariasi*) have been detected with respect to surveys conducted 15 and 17 years ago, respectively [16,17].

Clinical CanL shows a wide spectrum of clinical signs because of the many pathogenic mechanisms involved and the particular host immune response [18]. However, in areas where the disease is endemic a large number of infected dogs are asymptomatic (clinically healthy infected dogs) [19] but remain infective to sand flies [20,21]. An early diagnosis and effective treatment of sick dogs could help prevent the disease spreading to other dogs and/or humans.

In Europe, treatment generally consists of various dosage regimens of leishmanicides (mainly pentavalent antimonials or secondly, miltefosine) combined with allopurinol (a leishmanistatic drug) [22-25]. Allopurinol has proved highly effective at maintaining successfully treated dogs in long term clinical remission. However, these treatments are of questionable efficacy because most treated dogs are clinically cured but remain infective [20,26,27].

To determine the epidemiologic risks of CanL, the role of the dog as a natural reservoir for *L. infantum *and the capacity of the sand fly vector to transmit infected forms of *Leishmania *need to be addressed. To date, few studies have assessed the infection capacity of treated *L. infantum*-parasitized dogs through xenodiagnosis [20,26,28,29]. This is because the method is complex and colonies of phlebotomine vectors need to be kept in laboratory conditions throughout a complete life cycle of the insect [30,31].

This study was designed to assess through xenodiagnosis the infection capacity of dogs naturally infected with *L. infantum *before and after receiving three different treatment regimes, and to examine the clinical and parasite course.

## Methods

### Animals

This survey was carried out at the Veterinary Teaching Hospital of the Universidad Complutense de Madrid (Madrid, Spain). The dogs selected were 32 dogs infected with *L. infantum *that consecutively attended the clinic. The owners of the dogs enrolled were previously informed about the study protocol.

Dogs were included in the study if they met all the following criteria:

- at least two clinical signs consistent with CanL: asthenia and/or loss of weight, skin lesions, lymphoadenomegaly and/or splenomegaly, epistaxis - sporadic or persistent (unilateral or bilateral), ocular lesions, alterations in digestive function and/or joints.

- a positive indirect immunofluorescence antibody test (IFAT) for leishmaniosis-specific antibodies (cut-off 1:100).

- parasite observed in cultures of bone marrow aspirates grown in Novy-Nicolle-McNeal medium and/or promastigotes detected by direct xenodiagnosis.

Dogs with CanL were excluded if they fulfilled one of the criteria:

- concomitant infectious or vector-borne disease.

- pregnant or lactating female.

- severe renal, hepatic or cardiac failure.

- prior treatment with leishmanicides or corticosteroids.

### Sample collection

Dogs were examined four times throughout the study: before treatment (Day 0) and at 60, 120, and 180 days post-treatment (dpt) onset. On Day 0, dogs underwent an electrocardiogram to rule out heart disease. At each time point, blood, urine and bone marrow samples were collected and animals were scored for 13 clinical signs assessed in a physical examination using a categorized scoring system from 0 to 2 (Table 1).

**Table 1:** Scoring system used for the different clinical variables assessed before and after beginning treatment

CLINICAL SIGNS	0	1	2
Weight	normal	reduced	cachexia
Appetite	normal	reduced	anorexia
Behavior	normal	apathy	postration
Lymphoadenomegaly	absent	localized	generalized
Epistaxis	absent	moderate	severe
Cutaneous keratoseborrhea	absent	moderate	generalized
Ulcers	absent	simple	multiple
Onicogriphosis	absent	moderate	severe
Ocular lesions	absent	moderate	severe
Digestive disorders	absent	mild	severe
Arthropathy	absent	simple	multiple
Amyotrophy	absent	moderate	severe
Polyuria/Polydipsia	absent	moderate	severe

**CLINICAL-PATHOLOGICAL ABNORMALITIES**	**0**	**1**	**2**

Proteins	normal	elevated	reduced
A/G ratio	normal	reduced	-
Urea	normal	elevated	-
Creatinine	normal	elevated	-
ALT	normal	elevated	-

Complete blood count	0 = normal, 1 = anemia, 2 = leukocytosis, 5 = anemia + leukopenia, 7 = anemia + leukocytosis		

The clinical-pathological variables monitored were blood and urine data (urea, creatinine, ALT and protein electrophoresis). Additionally, the dogs were scored for 6 clinical-pathological abnormalities (Table 1). The IFAT for anti-*Leishmania*-specific immunoglobulin G (IgG) antibodies was performed as described previously [32] using the cut-off 1:100. Serum samples were also tested for the presence of anti-*Ehrlichia canis *IgG antibodies [33].

For each dog, we conducted selective culture of bone narrow aspirates on Novy-McNeal-Nicolle medium (NNN) and prepared three Giemsa-bone marrow smears to detect the presence of *L. infantum *amastigotes.

### Treatment protocol

Dogs were assigned to one of the three treatment groups:

Group A: Meglumine antimoniate and allopurinol

- 35 mg/kg of meglumine antimoniate given subcutaneously twice daily for 28 days.

- 10 mg/kg of allopurinol given orally twice daily for six months.

Group B: Meglumine antimoniate

- 35 mg/kg of meglumine antimoniate given subcutaneously twice daily for 28 days.

Group C: Allopurinol

- 10 mg/kg of allopurinol given orally twice daily for six months.

### Xenodiagnosis

The infectivity of dogs was assessed through direct xenodiagnosis. The local colony of *P. perniciosus *used in this study had been laboratory-reared at the Instituto de Salud Carlos III, Madrid [30]. The colony was kept in a chamber under controlled conditions of temperature (27-28°C), relative humidity (95-100%) and light cycle (17 hours light/7 hours dark).

Dogs were sedated by the intravenous injection of 0.5 mg/kg of medetomidine and their heads introduced into individual cube-shaped nets (50 cm wide × 50 cm high × 50 cm deep). Before treatment (Day 0) and 60, 120, and 180 dpt onset, dogs were exposed for one hour to 100 unfed, 7-day-old female sand flies released inside the nets. After one hour of exposure, the sand flies were carefully collected using a mouth aspirator. Fed flies collected from each dog were separated into individual nets (15 × 15 × 15 cm). Next the dogs were removed from their cages and intravenously administered 0.25 mg/kg of atipamezole.

Fed sand flies were kept inside the chambers for 5-7 days. Engorged females were dissected and the sand fly midgut was examined under a light microscope to detect *L. infantum *promastigotes.

### Outcome variables

The leismanicidal efficacy of treatment was assessed by examining bone marrow cultures over time. The infection capacity of the dogs was determined through xenodiagnosis at the different time points. The clinical course of the dogs was examined in each of the treatment groups by determining two indicators: percentage reduction in clinical score and percentage reduction in clinical-pathological abnormality score. Both of these indicators were calculated as: score Day 0 - score dpt/score Day 0).

### Statistical analysis

Quantitative data for the clinical signs and clinical-pathological abnormalities monitored are provided as medians and their corresponding interquartile ranges. Categorical variables are given as percentages. Baseline variables were compared among the groups using the Chi-squared test, or Fisher's exact test for categorical variables. The Kruskal-Wallis test was used to compare quantitative variables. To detect differences among groups and examine changes produced over time in the four outcome variables (leishmanicidal efficacy, infection capacity, percentage reduction in clinical signs and percentage reduction in clinical-pathological abnormalities), we constructed 4 longitudinal marginal models, one per outcome measure, using a generalized estimating equation (GEE) procedure. For leishmanicidal efficacy and infectivity to sand flies, marginal longitudinal models for binary data were used. Clinical sign and clinical-pathological abnormality percentage reductions were normalized by transforming these data into arc sen .

All analyses were performed using Stata v. 10.1 software (StataCorp LP, College Station, Texas, USA).

## Results

Table 2 shows the baseline characteristics of the dogs in the three treatment groups revealing no significant differences among them (p > 0.1). All dogs showed clinical-pathological abnormalities associated with the disease. Of the 32 dogs initially recruited, 6 were withdrawn from the study (2 from Group B and 4 from Group C) for several reasons: clinical failure, pregnancy, diabetes, intestinal obstruction, surgery or death. This left a final study population of 26 dogs; 9 females and 17 males aged from 9 months to 9 years. Of these dogs, 16 were pure breed and 10 were mongrels.

**Table 2:** Baseline characteristics of the dogs included in the three different treatment groups

	Group A	Group B	Group C
N	12	11	9

AGE (years), median (p25-p75)	4 (3-5)	3 (2-8)	5 (4-7)

PROT (g/dl), median (p25-p75)	8 (7.6-10.1)	7.2 (6.8-9.8)	8.5 (7.4-8.8)

A/G (g/dl), median (p25-p75)	0.44 (0.38-0.49)	0.46 (0.36-0.57)	0.43 (0.42-0.66)

UREA (mg/dl), median (p25-p75)	36 (29-45)	34 (26-48)	36 (30-46)

CREAT (mg/dl), median (p25-p75)	0.78 (0.72-0.87)	0.69 (0.59-0.9)	0.86 (0.66-0.98)

ALT (U/l), median (p25-p75)	67 (45-100)	57 (46-146)	89 (54-98)

CLIN SCORE, median (p25-p75)	6 (4-7)	6 (5-9)	6 (5-6)

ANALYTICAL SCORE, median (p25-p75)	5 (3-10)	3 (3-4)	3 (2-4)

IFAT, median (p25-p75)	800 (400-1600)	800 (800-1600)	400 (200-400)

CBC n, (%)^*^	9 (75)	4 (36)	5 (56)

^*^Percentage of dogs showing abnormal CBC (anemia, leukocytosis or leukopenia)

CBC: complete blood count; ALT: Alanine trnsaminase; A/G: Albumin/Globulin ratio

### Leishmanicidal efficacy

The results of the bone marrow cultures shown in Table 3 indicate a drop in the number of dogs positive for the parasite produced in response to treatment in all three groups. In Group A, the 8 dogs testing positive (67%) on Day 0 had dropped to 3 dogs (25%) at 180 dpt onset. In Group B, 9 dogs (100%) initially testing positive, dropping to 1 dog (11%) after 180 days of treatment and in Group C, the corresponding numbers were 4 (80%) and 1 (20%). Our marginal longitudinal models revealed the significant (p < 0.001) and similar (p > 0.1) nature of these reductions from Day 60 across all three groups.

**Table 3:** Bone-marrow culture and xenodiagnosis results recorded before and after starting treatment in each dog

Dog	Bone narrow culture				Xenodiagnosis			
	
	D0	D60	D120	D 180	D0	D60	D120	D 180
A1	+	+	+	+	+	-	+	-

A2	+	-	-	+	+	-	-	-

A3	-	-	-	-	+	-	-	-

A4	+	-	-	-	-	-	-	-

A5	+	-	-	-	-	-	-	-

A6	-	-	-	-	+	-	-	-

A7	+	-	-	-	+	-	-	-

A8	-	-	-	-	+	-	-	-

A9	+	-	-	-	-	-	-	-

A10	-	-	-	-	+	-	-	-

A11	+	-	-	-	+	+	-	-

A12	+	-	-	+	-	-	-	-

B1	+	-	-	-	+	-	-	-

B2	+	-	-	-	+	+	-	-

B3	+	-	-	-	+	-	-	-

B4	+	-	-	+	-	-	-	+

B5	+	-	-	-	+	-	-	-

B6	+	-	-	-	-	-	-	-

B9	+	-	-	-	-	-	-	-

B10	+	-	-	-	+	-	-	-

B11	+	-	-	-	+	-	-	-

C1	-	-	-	-	+	-	-	-

C3	+	+	+	+	+	-	-	-

C5	+	+	-	-	+	-	-	-

C7	+	-	+	-	-	-	-	-

C9	+	-	+	-	-	-	-	-

+ indicates positive culture result or infectious dog

D0, D60, D120 and D180 indicate the time points before treatment and after 60, 120 or 180 days of treatment respectively.

### Infectivity to sand flies

Our xenodiagnosis experiments also revealed a considerable drop in the infectivity of the differently treated dogs in all 3 groups (Table 3). Thus, in Group A, numbers of infectious dogs over the study period fell from 8 (67%) to no dogs. In Group B, numbers dropped from 6 (67%) to 1 (11%), and in Group C, from 3 (60%) to zero. Marginal models once again indicated that these reductions were both similar (p > 0.1) and significant (p < 0.001) after 60 days post-treatment onset in all three groups.

### Clinical efficacy

**Figure 1:**
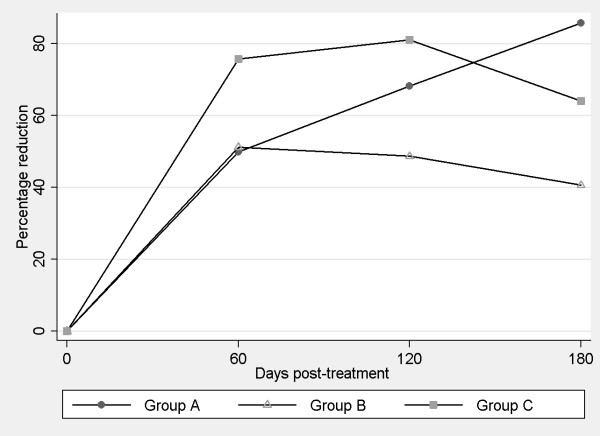
**Percentage reductions in clinical signs produced in response to treatment**.

Before the onset of treatment, the 26 dogs showed a median clinical score of 6 points. When the clinical course of disease was assessed in terms of the mean reduction in this score, significant differences were detected over time (p < 0.05) with respect to Day 0. When these reductions were compared among the groups, Group A treatment emerged as significantly more effective (p < 0.05) compared to the other two treatment regimens (Figure 1).

**Figure 2:**
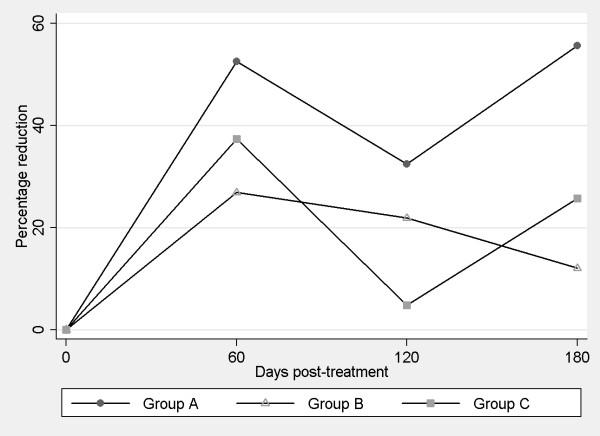
**Percentage reductions in clinicopathological abnormalities produced in response to treatment**.

Improved clinical-pathological abnormalities expressed as mean percentage reductions in scores observed indicated no significant differences (p < 0.05), improvements being observed only with respect to baseline (Day 0) scores. After treatment, significant differences were detected among the three groups such that the Group A treatment emerged as more effective (p < 0.05) than the remaining treatments (Figure 2).

## Discussion

To date, the use of xenodiagnosis to assess the efficacy of treatment, measured as the infection capacity of treated dogs naturally infected with *L. infantum*, has only been reported in four studies including 4, 2, 36 and 10 dogs respectively [20,26,28,29]. The present survey along with the study by Ribero et al. (2008) [28] was conducted on a sufficiently large sample size for valid conclusions to be drawn.

As may be observed in Figures 1 and 2, the most effective of our treatment regimens in terms of clinical improvement was the combined use of antimonials and allopurinol (Group A). Notwithstanding, we and other authors have noted that in some measure all treatments are able to improve the clinical condition of dogs [20,26,28,29].

By examining the capacity of the dogs to infect sand flies, we observed a considerable reduction in the infectivity of the dogs in response to the three treatments tested. Only 7.7% of the entire study population was able to infect sand flies after the second month of treatment. This finding suggests that similarly treated dogs will not play a major role in promoting or maintaining new foci of disease spread.

The liposome formulation of meglumine antimoniate has been described to significantly diminish the capacity of dogs to infect *Lutzomyia longipalpis *five months after the end of treatment [28]. In prior work, we observed a lack of infectivity towards sand flies of 6 dogs treated with meglumine antimoniate and allopurinol for at least 4 months post-treatment onset [20]. In early studies, treatment with meglumine antimoniate was found to reduce the percentage of sand flies becoming infected after feeding on treated dogs [26,29].

The bone marrow culture results indicate that regardless of a favorable clinical outcome of treatment, 30.8% of the dogs still showed the presence of the parasite in some of the check ups. These animals can be described as not parasitologically cured, or carriers of the parasite.

In the survey conducted in Brazil, bone marrow parasite burden was significantly reduced 4 days after the end of treatment with meglumine antimoniate [28]. In addition, it was observed that five of six dogs treated with meglumine antimoniate plus allopurinol still harbored parasites in the spleen 10 months after treatment [20].

It should be highlighted that in the treatment Group C of the present study, 80% of the dogs remained infected at the end of the study. This would appear to point to the ineffective nature of treatments based solely on allopurinol since, despite showing clinical improvement, these still infected animals could experience disease recrudescence. However, xenodiagnosis of the dogs in Group C indicated no evidence of the spread of the parasite to the phlebotomine sand flies. This finding has significant epidemiological implications in that although allopurinol may not be an effective shock treatment, it could perhaps be used in longer treatment maintenance courses (6-12 months). Notwithstanding, this should be done under supervision given the known long-term adverse effects of this agent (nephrolithiasis due to xanthine) [34,35].

The bone marrow culture and xenodiagnostic findings indicate that by Day 180 after the onset of treatment (A, B or C), 81% of the dogs had been "cured" or the parasite had been eliminated, although in some dogs (11.1 to 25%) it persisted for a variable length of time.

In conclusion, our results highlight the need to treat sick dogs since these represent a serious epidemiological risk. Despite not being a simple method, the xenodiagnostic approach used here emerged as a useful tool to assess the infection capacity of dogs treated with new drugs and/or new treatment regimens. A reduction in infectivity to sand flies in response to treatment is a key factor to consider in control programs designed to eradicate active foci of canine leishmaniosis. In endemic areas there is a plenty of evidence indicating the effectiveness of repellents against sand flies in reducing the spread of *Leishmania *infection.

## Competing interests

The authors declare that they have no competing interests.

## Authors' contributions

GM and RM designed, carried out the survey, drafted the first version of the manuscript and finalized the manuscript. RG helped with the xenodiagnosis experiments and finalized the manuscript. CF helped with the study design and carried out clinical experiments. MAD performed the statistical analysis of the data. All authors reviewed the manuscript.
